# Effects of topical application of resveratrol on tight junction barrier and antimicrobial compound production in lactating goat mammary glands

**DOI:** 10.1186/s13567-024-01276-z

**Published:** 2024-02-16

**Authors:** Yusaku Tsugami, Takahiro Nii, Naoki Isobe

**Affiliations:** 1 National Institute of Animal Health, National Agriculture and Food Research Organization, 4 Hitsujigaoka, Toyohira, Sapporo, Hokkaido 062-0045 Japan; 2 Graduate School of Integrated Sciences for Life, Hiroshima University, 1-4-4 Kagamiyama, Higashi-Hiroshima, Hiroshima 739-8528 Japan

**Keywords:** Antimicrobial compound, mammary gland, resveratrol, tight junction

## Abstract

In mammary glands, the formation of less-permeable tight junctions (TJs) and the production of antimicrobial compounds like lactoferrin and defensins are important for preventing mastitis. Resveratrol, a polyphenol contained in red grapes, is known to protect mammary epithelial cells (MECs) from oxidative stress; however, oral administration of resveratrol causes a decrease in certain biological processes through conjugation and metabolic conversion. In this study, we determined the beneficial effects of resveratrol on TJs and antimicrobial compounds in cultured goat MECs by adding it to the medium, and in lactating goat mammary glands by topical application for percutaneous absorption. TJ barrier function was evaluated by transepithelial resistance and expression or localization pattern of claudins for culture model in vitro and by somatic cell count, Na^+^, albumin, and IgG in milk for topical application in vivo. Concentrations of antimicrobial compounds and cytokines were measured using ELISA. Activation of STAT3 was evaluated by Western blotting. Resveratrol strengthened TJ barrier function by upregulating claudin-3 in cultured MECs and topical application to udders reduced somatic cell count, Na^+^, albumin, and IgG in milk. Resveratrol increased β-defensin and S100A7 levels in cultured MECs and milk. In addition, resveratrol down-regulated cytokine production and STAT3 pathway. These findings suggest that the topical application of resveratrol to udders may be effective in preventing mastitis.

**Supplementary Information:**

The online version contains supplementary material available at 10.1186/s13567-024-01276-z.

## Introduction

Mastitis results in large economic losses in the milk production industry including increased treatment costs [1]. Although antibiotic administration is the main treatment for mastitis, the reduction of antibiotic use is recommended due to the risk of developing antimicrobial resistance in bacteria [2]. In order to reduce antibiotic use, the first step is to prevent the onset of mastitis. The common prevention for mastitis is maintaining a clean environment and milking management. Several vaccinations are also used for prevention, although its effectiveness is debatable and dairy cows must be vaccinated by a qualified veterinarian. Therefore, new and simple methods need to be established to prevent mastitis. Mastitis is caused by pathogens that invade the teat canals [3]. In mammary glands, there is a defense system against mastitis-causing pathogens. One is formation of less-permeable tight junctions (TJs) [4], and the other involves the production of antimicrobial compounds like lactoferrin and defensins [5].

TJs are composed of occludin and claudin, which are transmembrane proteins [6]. In the luminal epithelium, such as the intestines, lungs, and mammary glands, TJs are formed at the most-apical regions in the lateral membranes. TJs regulate the paracellular permeability of water, ions, and small molecules [7]. The specific subtype of claudins at TJ regions determines permeability [8]. In mammary epithelial cells (MECs), the expression and localization of claudin-3 and claudin-4 are altered depending on the stage of pregnancy, lactation, and involution [9]. In lactating MECs of both rodents and ruminants, claudin-3 localizes to TJ regions and contributes to the formation of less-permeable TJs known as the blood-milk barrier [10, 11]. The less-permeable TJs prevent blood components from leaking into milk, regulate the migration of leukocytes into mammary alveoli, and prevent pathogens from invading the body [4]. In contrast, in mammary glands affected by mastitis or during involution, increased amounts of claudin-4 localize to TJ regions, which causes leakage [12].

In lactating mammary glands, various antimicrobial compounds are secreted into milk [5]. The antimicrobial mechanism against pathogens depends on the type of antimicrobial compound. Antimicrobial activity of defensins and cathelicidins are characterized by disruption of the integrity of the microbial cell membrane [13, 14]. Lactoferrin has a bacteriostatic effect decreasing iron availability to pathogens [15]. S100A7 and S1008A is a S100 protein family, which has EF-hand helix–loop–helix domains for calcium-binding [16]. S100A7 has an antimicrobial activity against *Escherichia coli* (*E. coli*) [17]. S100A8 is a subunit of calprotectin and sequesters essential trace metals from microbes, which inhibits growth [18]. IgA and IgG are responsible for bacterial agglutination and toxin neutralization [19]. These antimicrobial compounds are produced by MECs and leukocytes in the mammary glands. Lactoferrin and β-defensin-1 are produced by MECs and leukocytes [20, 21]. S100A7 is primarily produced by epithelial cells [22], whereas S100A8 and cathelicidin-2 are mainly produced by leukocytes [23, 24]. IgA and IgG are produced by plasma cells differentiated from B cells and migrate into milk through polymeric immunoglobulin receptors in MECs or through paracellular pathways [19, 25].

Resveratrol is found in grapes and their products, such as wine, at high concentrations. The amount of resveratrol varies between wine type since resveratrol is mainly found in the skin of red grapes: red wine contains more than 580 μg per 100 mL, whereas white wine contains less than 68 μg per 100 mL [26]. Resveratrol has two isomeric forms: trans-resveratrol and cis-resveratrol. Although both forms exhibit biological activity, the majority of resveratrol biological effects are attributable to trans-resveratrol, which is the more stable form [27]. Resveratrol has been reported to protect bovine MECs in vitro from oxidative damage induced by H_2_O_2_ [28]. In TJs, resveratrol ameliorates intestinal barrier damage and inflammation caused by dextran sodium sulfate or deoxynivalenol [29, 30]. Resveratrol also protects the blood–brain barrier in experimental autoimmune encephalomyelitis mice [31] and restores decreased claudins following damage to the intestines and brain. With respect to antimicrobial compound production, resveratrol or its precursor, polydatin, enhance the production of β-defensins induced by heat-stress or *Streptococcus pneumoniae* in lung cells or keratinocytes [32, 33].

The oral administration of polyphenols often causes a decrease in biological activities at target organs through conjugation and metabolic conversion. Free resveratrol in the blood reaches a peak at 30 min after ingestion by gastric absorption, but the maximum concentration of free resveratrol is extremely low and less than 37 nM in humans [34]. Ingested resveratrol is rapidly metabolized and several metabolites are detected in the blood, such as resveratrol-O-glucuronides or resveratrol-O-sulfates [35]. Some studies suggest that resveratrol does not exert significant biological activity because of its rapid conjugation and metabolism following oral administration [36–38]. In this study, we focused on the percutaneous absorption of resveratrol. The molecular weight of resveratrol is 228, which allows transdermal absorption [39]. Therefore, we hypothesized that its topical application to udders could influence the TJ barrier and antimicrobial compound production in mammary glands through percutaneous absorption to avoid conjugation and metabolism. We determined the effects of resveratrol on TJs and antimicrobial compounds in cultured goat mammary epithelial cells (GMECs) in vitro by adding it to the medium, and in vivo by applying it topically to lactating goat mammary glands.

## Materials and methods

### Experimental design

First, we investigated the effects of resveratrol on in vitro GMECs. According to previous studies that have employed cultured MECs and resveratrol [28, 40], GMECs were exposed to resveratrol at concentrations ranging from 0.5 to 50 μM for 3 days. Additionally, as the effects of polyphenol often change with prolonged treatment [41], we performed long-term treatment using 10 μM resveratrol for 9 days. In case of 1 day treatment, GMECs were cultured without resveratrol for 8 days and it was added only on the last day. For 3 day treatment, resveratrol was not included for 6 days and was introduced only 3 days before sample collection. The control group was maintained in a normal differentiation medium with 0.1% dimethyl sulfoxide (DMSO) without resveratrol for 9 days.

Since a previous in vivo study demonstrated that gingerol, a ginger polyphenol, exhibits the same effects as in vitro experiments with antimicrobial compounds when applicated at 100 μM for 7 days [42], we also conducted an in vivo experiment under the given conditions. Moreover, there are variations in each parameter of milk components depending upon the udder even in the same individual [43]. Therefore, we compared the changes in milk component values before and after treatment within the same udder.

The sample size was determined based on a previous study [42], with *n* = 8–12 for in vitro experiments and *n* = 9 for in vivo experiments.

### Animals

Nine healthy Tokara goats (mid-lactation stage, milk yield 38–240 mL/udder/day, 21.6–33.6 kg, 2–4 years, 1–3 parity) were used for this in vivo experiment. The goats were individually housed under a 14:10 h light: dark cycle, fed 0.6 kg hay cubes and 0.2 kg barley each day, and had free access to water and trace-mineralized salt blocks. Feed was offered twice daily at 08:00 and 15:00 h. The feed supply (energy, protein, and minerals) was calculated based on the Japanese feeding standard for sheep (Ministry of Agriculture, Forestry and Fisheries in Japan, 1996) as reported previously [44]. Milking was performed by hand once daily at 08:00 h. None of the 9 goats had mastitis (no clinical symptoms and SCC less than one million cells/mL [45]). All experiments were approved by the Animal Research Committee of Hiroshima University (no. C21-20) and conducted according to the Guidelines for Animal Experiments prescribed by Hiroshima University.

### Culture of GMECs

We established a MEC culture model based on a previously described model [11, 46] with some modifications. Briefly, mammary gland tissues were collected from Tokara goats at mid-lactation as previously described [47] with some modifications. Deep sedation and anesthesia were achieved by slow intravenous injection of xylazine (Bayer HealthCare Pharmaceuticals Inc., Leverkusen, Germany) and pentobarbital (Somnopentyl; Kyoritsu Seiyaku, Tokyo, Japan), and the goats were euthanized by exsanguination. Primary GMECs were isolated from minced mammary glands using collagenase and trypsin. Cell selection was achieved by centrifugation at 20 × *g* for 5 min with Dulbecco Modified Eagle Medium/Ham F-12 (DMEM/F12) medium (Sigma-Aldrich, St. Louis, MO, USA) containing 60% fetal bovine serum (MP Biomedicals, Irvine, CA, USA). The isolated GMECs were stored in Bambanker (Wako, Osaka, Japan) at −80 °C until use.

Defrosted GMECs were cultured in 24-well plates or 12-well cell culture inserts (0.4 µm pore size; BD Biosciences, Bedford, MA) coated with collagen gel (Cellmatrix type 1A; Nitta Gelatin, Osaka, Japan) with growth medium consisting of DMEM/F12 medium supplemented with 5% fetal bovine serum, 5 µg/mL ITS-X (1 mg/mL insulin, 0.55 mg/mL transferrin, 0.67 mg/mL selenium, 0.20 mg/mL ethanolamine; Wako), 10 ng/mL epidermal growth factor (BD Biosciences), and 5 mM sodium acetate (Nacalai Tesque, Kyoto, Japan) for 6 days until confluent. Subsequently, the GMECs were cultured in a differentiation media containing 1% fetal bovine serum, 5 µg/mL ITS-X, 1 ng/mL epidermal growth factor, 5 mM sodium acetate, 1 μg/mL prolactin (provided by A F Parlow, lot AFP7170E, NHPP, NIDDK, Torrance, CA, USA), and 1 µM dexamethasone (Sigma-Aldrich) in DMEM/F12 media. The upper chamber of the insert was filled with Hanks Balanced Salt Solution (HBSS) (Thermo Fisher Scientific, Waltham, MA, USA) for differentiation. After 2 days of culture, the GMECs were treated with resveratrol or 0.1% DMSO as a vehicle control. Resveratrol was purchased from the Tokyo Chemical Industry (#R0071, Tokyo, Japan) and dissolved in DMSO.

To measure transepithelial resistance, the electrodes of a Millicell-ERS-2 (Millipore, Billerica, MA, USA) were placed in the upper and lower chambers of the insert and the resistance was measured.

### Topical application to udders

To determine the effects of resveratrol on the mammary glands of lactating goats, 1 mL of 100 μM resveratrol in 70% ethanol containing 0.2% DMSO was applied to the surface of the udders on days 0–6 after milking at 08:00 h. As a placebo, 70% ethanol containing 0.2% DMSO was applied to another udder (Additional file 1). Milking was completed within 10 min, and application was completed within 2 min in the order of placebo udder and resveratrol udder.

### Analysis of milk samples

Milk samples were centrifuged at 1000 × *g* for 10 min at 4 °C. Milk fat and skim milk were separated from the somatic cell pellets. The pellets were resuspended with PBS and centrifuged at 1000 × *g* for 10 min at 4 °C. After repeating the process twice, the cell pellets were resuspended in PBS to determine the somatic cell count (SCC), which was measured with a Countess II FL Automated Cell Counter (Thermo Fisher Scientific) as described previously [48]. Skim milk was stored at −30 °C for later enzyme-linked immunosorbent assay (ELISA). The Na^+^ concentration was measured using a LAQUAtwin Na-11 pocket meter (HORIBA, Kyoto, Japan).

### ELISA

Competitive ELISA was performed to measure lactoferrin, β-defensin-1, cathelicidin-2, cathelicidin-7, S100A7, S100A8, interleukin (IL)-1β, IL8, and tumor necrosis factor (TNF)-α, as described previously [24, 48, 49]. Briefly, a rabbit was immunized with synthetic peptides against the target proteins (Scrum, Tokyo, Japan). After whole blood collection, antibodies were purified using a HiTrap Protein G High Performance affinity column (Cytiva, Tokyo, Japan). The purified antibodies were coated on the ELISA plate. The target peptides were conjugated with horseradish peroxidase (HRP) using a Peroxidase Labeling Kit-NH2 or Peroxidase Labeling Kit-SH (Dojindo Laboratories, Kumamoto, Japan). For lactoferrin, anti-lactoferrin antibody (Life Laboratory Company, Yamagata, Japan), lactoferrin purified from goat milk, and lactoferrin conjugated with HRP were used.

Sandwich ELISA was performed to measure albumin, IgG, and IgA levels using an anti-albumin antibody (Life Laboratory Company), goat-albumin antibody-HRP (#A50-103P, Bethyl Laboratories, Montgomery, TX), goat-IgA antibody (#A50-106A, Bethyl Laboratories), goat-IgA antibody-HRP (#A50-106P, Bethyl Laboratories), goat-IgG antibody (#A50-104A, Bethyl Laboratories), and goat-IgG antibody-HRP (#A50-104P, Bethyl Laboratories).

After washing with PBS, GMECs cultured on plates were lysed in radioimmunoprecipitation buffer (25 mM Tris–HCl [pH7.6], 150 mM NaCl, 1% NP-40, 1% sodium deoxycholate, 0.1% sodium dodecyl sulfate, and protease inhibitors). Sample dilutions yielding optical density readings in the linear portion of a standard curve were used to quantify the level of each protein. Standard and sample dilutions were added to each ELISA plate in duplicate, and the optical density was measured with a microplate reader (Multiskan FC Microplate type 357; Thermo Fisher Scientific). Intracellular protein concentrations of the GMECs measured by ELISA were normalized to the total protein concentration. The ELISA performed in this study was specific for goats.

### Fractionation of triton X-100-soluble and triton X-insoluble proteins

To determine the amount of claudins at TJ regions, GMECs were separated into Triton X-100-soluble and insoluble fractions as previously reported [50], with some modifications. GMECs cultured in inserts were lysed in Triton X buffer (0.1% Triton X, 100 mM NaCl, 10 mM HEPES at pH 7.4, 2 mM EDTA and protease inhibitors) and passed 30 times through a 20-gauge needle. The lysate was centrifuged at 20 000 × *g* for 30 min at 4 °C. The supernatant was considered as the Triton X 100-soluble fraction, and the pellet was considered as the insoluble fraction.

### Western blot analysis

Western blot analysis was performed as previously described [51], with some modifications. Samples were lysed in a Laemmli sodium dodecyl sulfate-solubilizing buffer and heated for 15 min at 70 °C. The samples were separated on sodium dodecyl sulfate–polyacrylamide gels and transferred to polyvinylidene difluoride membranes (Bio-Rad Laboratories, Hercules, CA, USA). Signals were detected using claudin-3 (#34-1700, Thermo Fisher Scientific), claudin-4 (#PA5-32354, Thermo Fisher Scientific), α-tubulin (#GTX628802, GeneTex, Los Angeles, CA, USA), pSTAT3 (#sc-8059, Santa Cruz Biotechnology, Santa Cruz, CA, USA), STAT3 (#sc-8019, Santa Cruz Biotechnology), secondary HRP-conjugated anti-rabbit antibody (Abcam, Cambridge, UK), anti-mouse antibody (Sigma-Aldrich), and the Immobilon Forte Western HRP Substrate (Millipore). Band images were obtained using the Ez-Capture II (Atto, Tokyo, Japan). The bands were quantitated and analyzed using a CS Analyzer 3.0 (Atto).

### Immunofluorescence

Immunofluorescence was performed, as previously described [50], with some modifications. GMECs in a collagen gel isolated from the insert were fixed with methanol for 10 min at −20 °C followed by 1% paraformaldehyde in PBS for 10 min at 4 °C. The following antibodies were used for immunofluorescence: claudin-3 (#34-1700), occludin (#sc-133256, Santa Cruz Biotechnology), and secondary antibodies (Alexa Fluor 488-conjugated goat anti-rabbit, #A32731; Alexa Fluor 555-conjugated goat anti-mouse, #A32727; Thermo Fisher Scientific). Images were obtained using a fluorescence microscope (BZ-9000) and processed using analysis software (Keyence, Osaka, Japan).

### Quantitative polymerase chain reaction (qPCR)

Quantitative PCR was performed as previously described [52]. Total RNA from GMECs cultured on plates was extracted using Sepasol-RNA I Super G (Nacalai Tesque). Reverse transcription was performed using the ReverTra Ace qPCR RT Master Mix (Toyobo, Osaka, Japan). Quantitative PCR was conducted on an AriaMx Real-Time PCR System (Agilent technologies, Santa Clara, CA, USA) with Brilliant III Ultra-Fast SYBR QRTPCR (Agilent technologies). The cycling conditions were 95 °C for 1 min followed by 45 cycles at 95 °C for 15 s and 60 °C for 1 min. The primers for *BD1* (β-defensin-1), *S100A7*, and *LTF* (lactoferrin) are listed in Table 1. Ribosomal protein S18 (*RPS18*) was used as an internal control, which was selected by BestKeeper among RPS18, actin beta (*ACTB*), and glyceraldehyde-3-phosphate dehydrogenase (*GAPDH*).

**Table 1:** **Primer sequences for quantitative PCR**

Gene	Accession number	Primer	Product size
*BD1*	XM_018042143.1	(F) ACTCAAGGAATAAGAAGTCG	116
		(R) CATTTTACTGGGGGCCCGAA	
*LTF*	NM_001285548.1	(F) CGCAGTGTGGATGGCAAGGAG	215
		(R) TTCAAGGCGGTCAAGTAACGG	
*S100A7*	NM_174596.2	(F) GAAGCCAAGATGAGCAGCTCTC	329
		(R) GGAGGCCTCTGGGCTCACT	
*GAPDH*	XM_005680968.3	(F) CCTGGAGAAACCTGCCAAGT	200
		(R) GCCAAATTCATTGTCGTACCA	
*ACTB*	NM_173979.3	(F) CATCACCATCGGCAATGAG	151
		(R) CCGTGTTGGCGTAGAGGTC	
*RPS18*	NM_001285639.1	(F) TAATCCCGCCGAACCCCATT	125
		(R) GGTGTGTACAAAGGGCAGG	

### Statistical analysis

**Figure 1:**
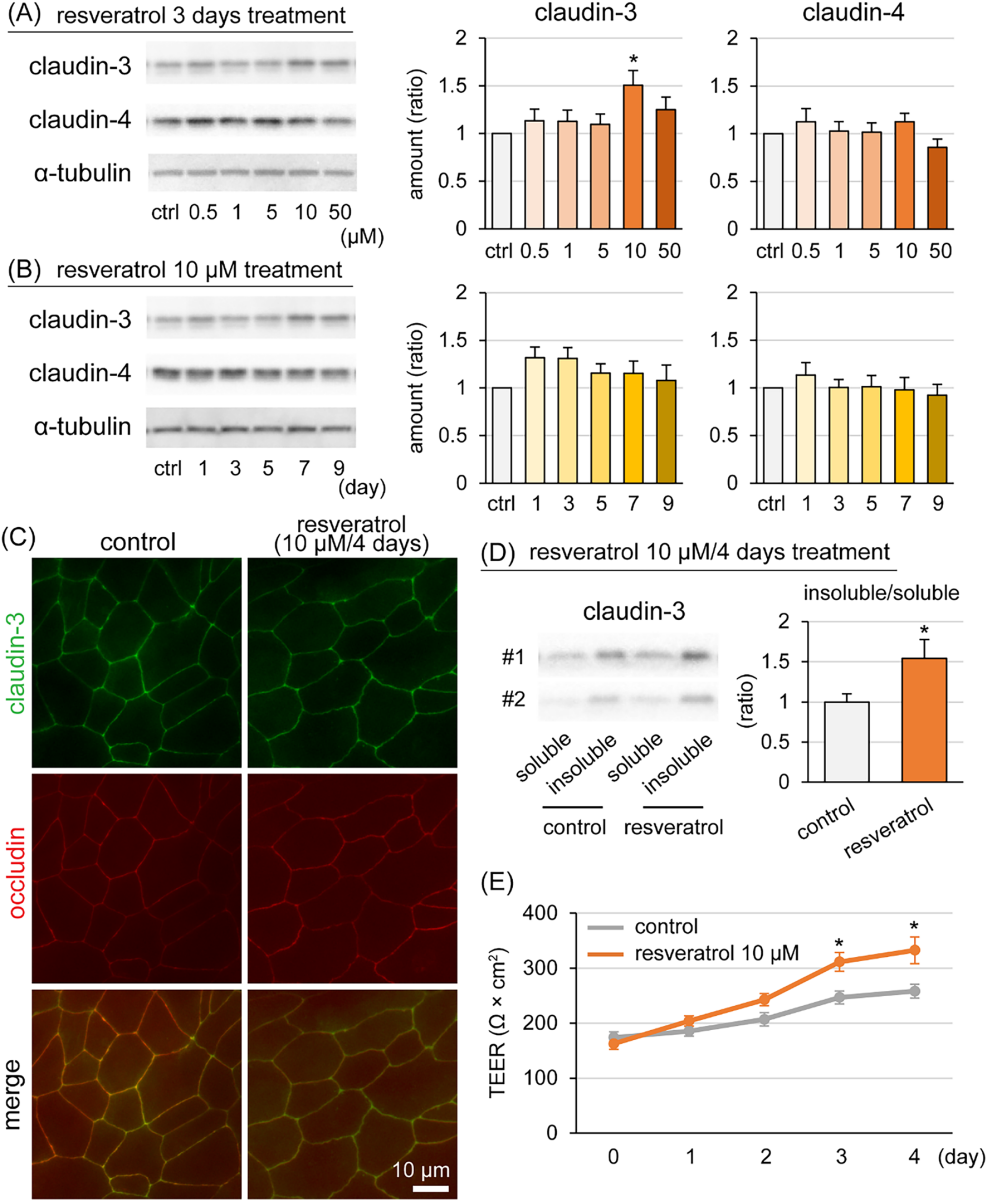
**Effect of resveratrol on the tight junction barrier of goat mammary epithelial cells (GMECs).** Western blot analysis of claudin-3 and claudin-4 in GMECs treated with resveratrol at various concentrations for 3 days (**A**) or treated with 10 μM resveratrol each day (**B**). The graphs show the results of densitometry analysis; α-tubulin was the internal control. **C** The images show the localization of claudin-3 (green) and occludin (red; tight junction marker) in GMECs treated with 10 μM resveratrol for 4 days. Scale bar 10 µm. **D** Western blot analysis of Triton X-100-soluble and -insoluble fractions of claudin-3. The graph shows the ratio of insoluble/soluble claudin-3. **E** The graph shows transepithelial resistance in GMECs. Control GMECs were treated with 0.1% dimethyl sulfoxide. The data are presented as the mean ± SEM (*n* = 8–12). Asterisks show significant differences (*p* < 0.05 versus control).

**Figure 2:**
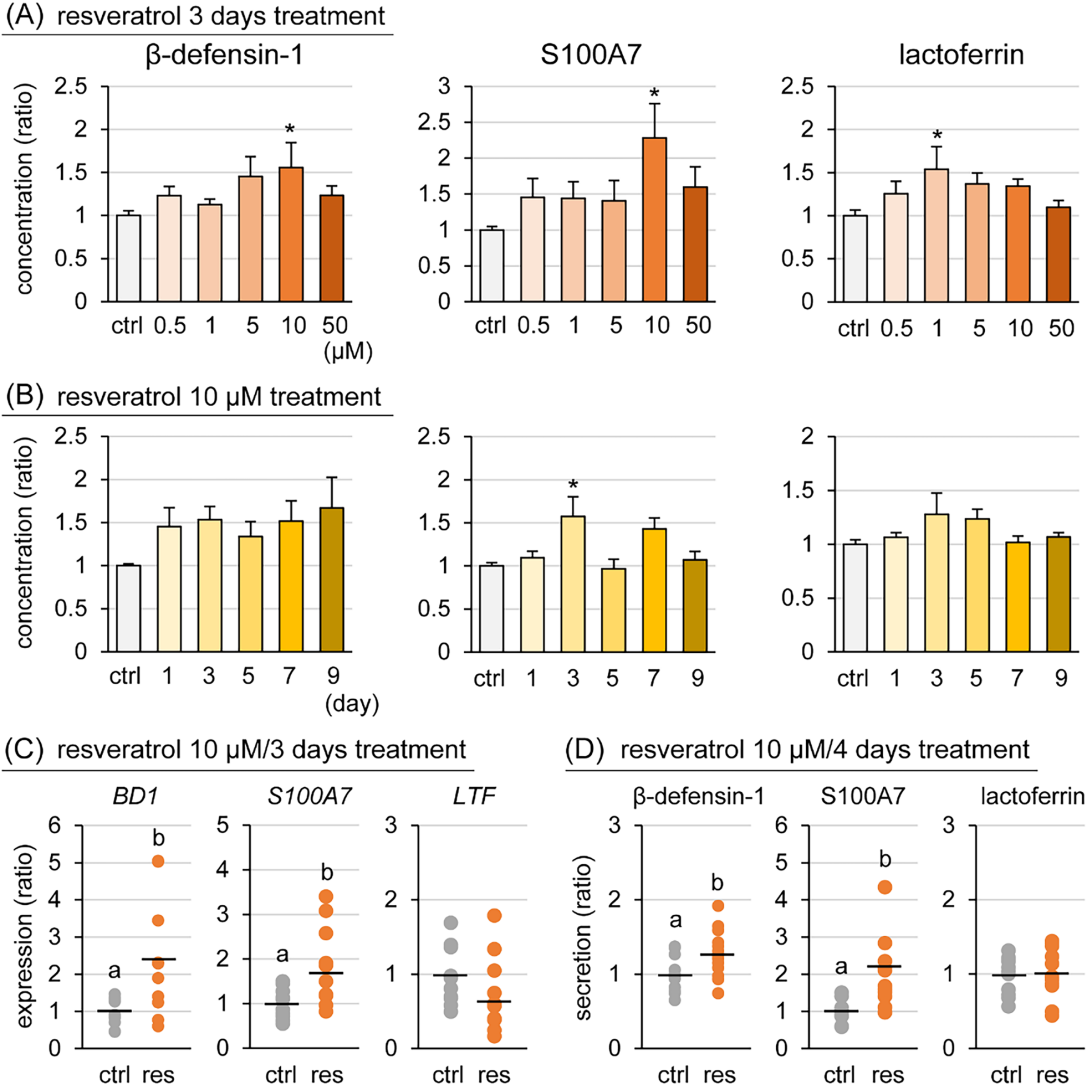
**Effect of resveratrol on antimicrobial compound production in goat mammary epithelial cells (GMECs).** β-defensin-1, S100A7, and lactoferrin levels as measured by ELISA in GMECs treated with resveratrol at various concentrations for 3 days (**A**) or treated with 10 μM resveratrol each day (**B**). **C** The expression of *BD1* (β-defensin-1), *S100A7*, and *LTF* (lactoferrin) mRNA was detected using qPCR in GMECs treated with 10 μM resveratrol for 3 days. **D** β-defensin-1, S100A7, and lactoferrin levels as measured by ELISA in the medium of the upper chamber of inserts treated with 10 μM resveratrol for 4 days. Control GMECs were treated with 0.1% dimethyl sulfoxide. The data are presented as the mean ± SEM (*n* = 8–12). Asterisks show significant differences (*p* < 0.05 versus control).

Data are expressed as the mean ± SEM. Statistical analyses were performed using SAS software (version 9.4, SAS Institute Inc., Cary, NC, USA). Significance values were calculated using a one-way analysis of variance and post-hoc Student *t*-test (Figures 1D, E, 2C, D) or Dunnett test (other). Differences were considered statistically significant at *p*-values < 0.05. The in vivo experiments were performed using nine different udders and the in vitro experiments were performed using three different GMECs derived from different goats. In the in vitro experiments, the number of samples was defined as *n* = 1 for one sample obtained from one well. The relative values of milk yield, SCC, and other components were calculated based on the average pretreatment levels (days −1 and 0). Western blot analysis using one acrylamide gel with 2 or 3 sets of GMECs samples originating from different well plates or cell culture inserts was performed 4 to 6 times. For quantification of the bands in GMECs treated with resveratrol, depending on concentration or time, the relative value of 1 set of GMECs samples was calculated based on each control. Experiments were performed in duplicate or triplicate.

## Results

### The influence of resveratrol on TJs in GMECs

GMECs were treated with resveratrol (0.5, 1, 5, 10, or 50 μM) or 0.1% DMSO as a vehicle control for 3 days and the amount of claudin-3 and claudin-4 was determined by Western blot analysis (Figure 1A). Resveratrol treatment at 10 μM significantly increased the level of claudin-3 to 1.5-fold that of the control group, while no significant difference was observed for claudin-4. In order to investigate long-term treatment effects of resveratrol, GMECs were treated with resveratrol at 10 μM for 9 days, but there were no statistical differences in the amount of claudin-3 or claudin-4 (Figure 1B). The localization of claudin-3 was determined by immunofluorescence (Figure 1C). In the control group, claudin-3 was co-localized with occludin, which is a marker of TJ regions. GMECs treated with 10 μM resveratrol for 4 days also exhibited the same localization as the control group. The subcellular localization of claudins was also examined by isolating the Triton X-100-soluble and insoluble fractions. The Triton X-100-soluble fractions primarily contained claudins in the cytosol and in the lateral membranes, whereas claudins in the Triton X-100-insoluble fractions originated from TJ strands [53]. Resveratrol treatment at 10 μM for 4 days significantly increased the ratio of Triton X-insoluble/soluble claudin-3 to 1.5-fold compared with the control group (Figure 1D). The effect of resveratrol on epithelial barrier function was determined by measuring transepithelial resistance (Figure 1E). Resveratrol treatment at 10 μM showed higher transepithelial resistance in the MEC layer compared with the control group after a 3- and 4-day treatment.

### Effect of resveratrol on antimicrobial compound production in GMECs

GMECs were treated with resveratrol (0.5, 1, 5, 10, or 50 μM) or 0.1% DMSO for 3 days and the concentration of β-defensin-1, S100A7, and lactoferrin in GMECs was determined using ELISA (Figure 2A). Resveratrol treatment at 10 μM resulted in a significantly higher intracellular concentration of β-defensin-1 and S100A7 (1.5- and 2.2-fold, respectively) compared with the control group. In addition, 1 μM resveratrol significantly increased lactoferrin in GMECs to approximately 1.5-fold that of the control group. GMECs were treated with resveratrol at 10 μM for 9 days and resveratrol treatment for 3 days increased the intracellular concentration of S100A7, while no significant difference was observed for β-defensin-1 and lactoferrin (Figure 2B). The effects of resveratrol on mRNA expression were examined by qPCR (Figure 2C). GMECs treated with 10 μM resveratrol for 3 days show significantly higher *BD1* (β-defensin-1) and *S100A7* expression (2.2- and 1.8-fold, respectively) compared with that of the control group (*p* < 0.05). To determine the effect of resveratrol on the secretion of antimicrobial compounds, GMECs were treated with resveratrol at 10 μM for 4 days and the concentrations of the components in the medium of the upper chamber were determined by ELISA (Figure 2D). Resveratrol treatment resulted in significantly higher concentrations of β-defensin-1 and S100A7 in the culture medium (1.2- and 2.1-fold, respectively) compared with the control group.

### Effect of topical resveratrol application to udders

**Figure 3:**
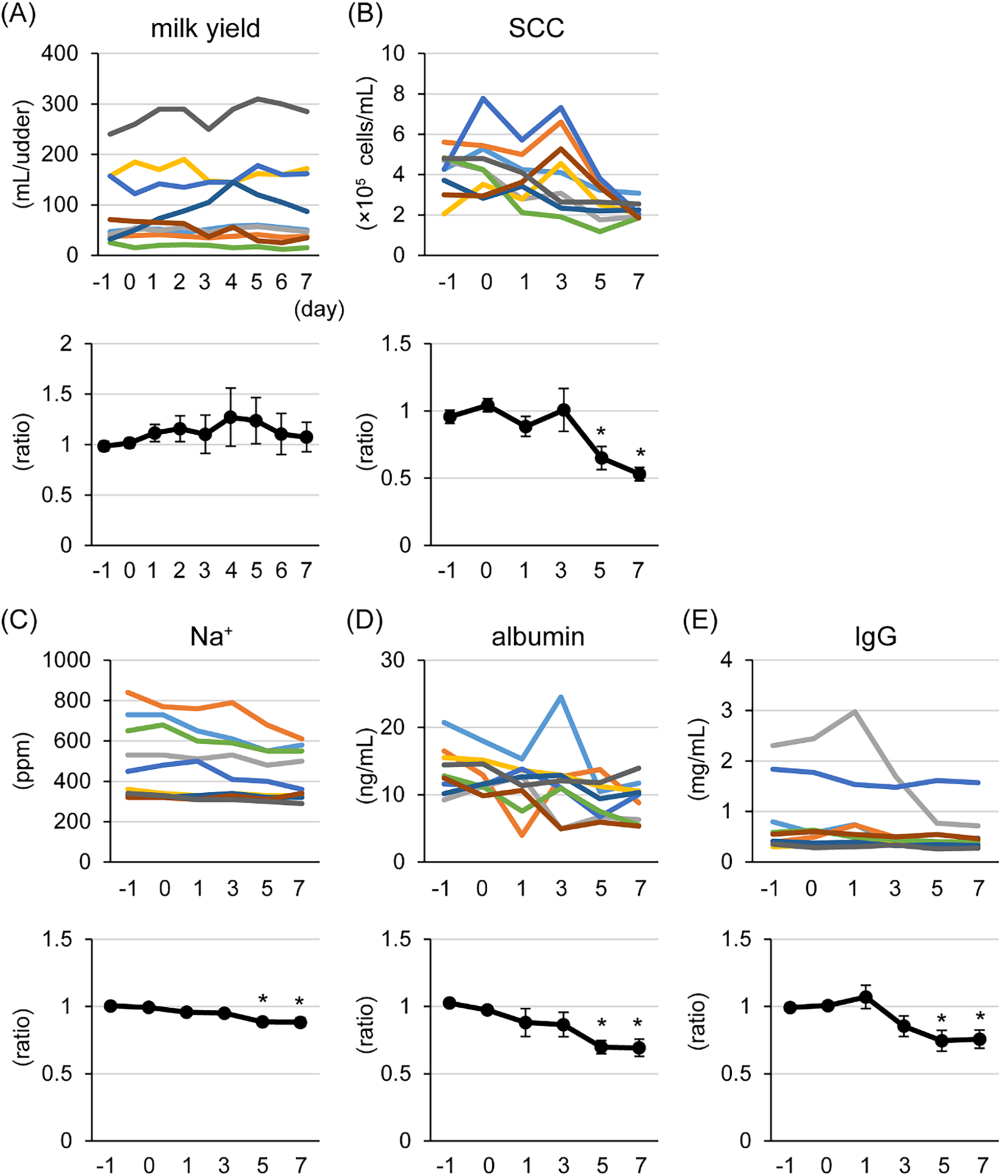
**Influence of a topical application of resveratrol to udders on milk yield, somatic cell count (SCC), and blood-derived components in milk.** The udders of lactating goats were topically treated with 1 mL of 100 μM resveratrol in 70% ethanol containing 0.2% dimethyl sulfoxide for 7 days. The upper graphs show changes in milk yield (**A**), SCC (**B**), Na^+^ (**C**), albumin (**D**), and IgG (**E**) in individual udders and the lower graphs show changes in the relative values compared with the average of the pre-treated (days −1 and 0) controls. The data are presented as the mean ± SEM (*n* = 9). Asterisks show significant differences (*p* < 0.05 versus control).

**Figure 4:**
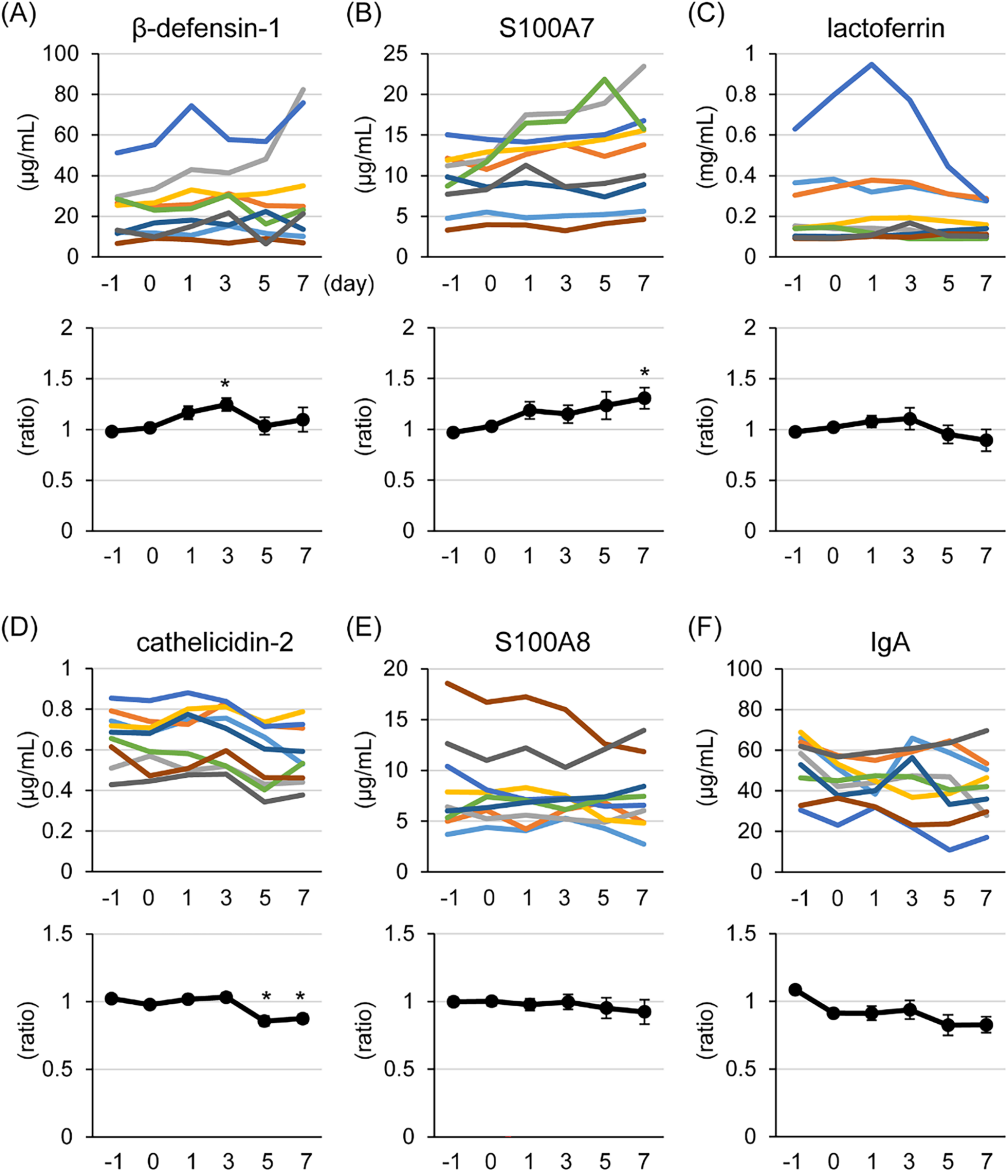
**Influence of a topical application of resveratrol to udders on antimicrobial compound concentration in milk.** The udders of lactating goats were topically treated with 1 mL of 100 μM resveratrol in 70% ethanol containing 0.2% dimethyl sulfoxide for 7 days. The upper graphs show changes in β-defensin-1 (**A**), S100A7 (**B**), lactoferrin (**C**), cathelicidin-2 (**D**), S100A8 (**E**), and IgA (**F**) levels in individual udders and the lower graphs show the changes in the relative values compared with the average of pre-treated (days −1 and 0) controls. The data are presented as the mean ± SEM (*n* = 9). Asterisks show significant differences (*p* < 0.05 versus control).

One mL of 100 μM resveratrol in 70% ethanol containing 0.2% DMSO was applied to the udders of lactating goats for 7 days. The SCC and concentrations of Na^+^, albumin, and IgG in the milk were significantly decreased following treatment for 5 and 7 days compared with the average at day −1 and 0 (pre-treatment; control) (Figure 3). There were no statistical differences in milk yield. The concentration of antimicrobial compounds in milk were measured by ELISA (Figure 4). β-defensin-1 and S100A7 were present at significantly higher levels following resveratrol treatment for 3 days and 7 days, respectively, compared with that of pre-treated controls. In contrast, the concentration of cathelicidin-2 in milk was significantly lower following treatment for 5 and 7 days compared with that of pre-treatment controls (Figure 4D).

### Effect of resveratrol on cytokine production

**Figure 5:**
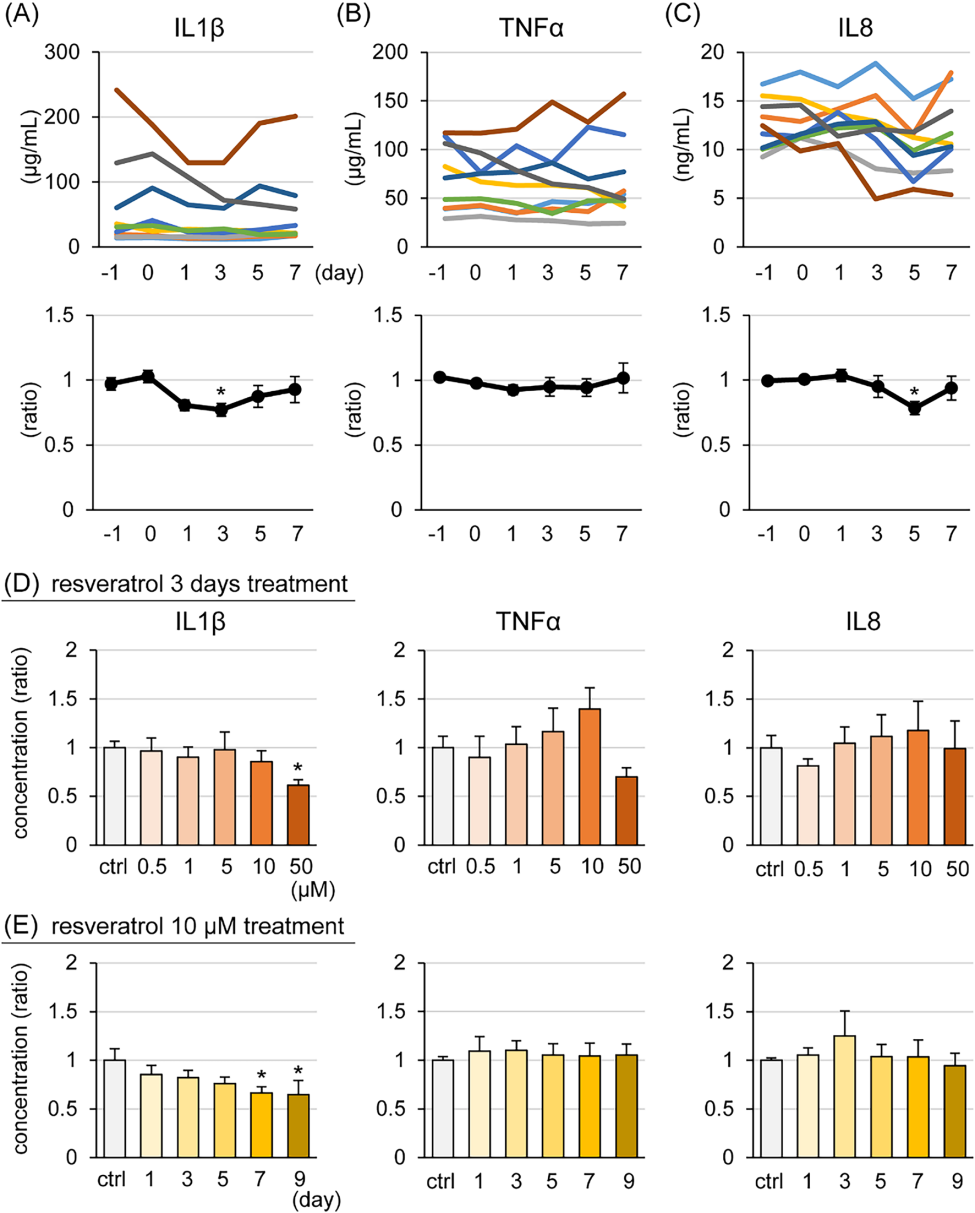
**Effect of resveratrol on cytokine production in goat mammary epithelial cells (GMECs) and concentration in milk.** The udders of lactating goats were topically treated with 1 mL of 100 μM resveratrol in 70% ethanol containing 0.2% dimethyl sulfoxide for 7 days. The upper graphs show the changes in IL1β (**A**), TNFα (**B**), and IL8 (**C**) levels in individual udders and the lower graphs show the changes in the relative values compared with the average of pre-treated (days −1 and 0) controls. The data are presented as the mean ± SEM (*n* = 9). IL1β, TNFα, and IL8 levels as measured by ELISA in GMECs treated with resveratrol at various concentrations for 3 days (**D**) or treated with 10 μM resveratrol each day (**E**). Control GMECs were treated with 0.1% dimethyl sulfoxide. The data are presented as the mean ± SEM (*n* = 8). Asterisks show significant differences (*p* < 0.05 versus control).

The concentration of cytokines in milk were measured by ELISA (Figure 5A). IL1β and IL8 levels were significantly lower following treatment for 3 days and 5 days, respectively, compared with that in pre-treatment controls. In addition, GMECs treated with 50 μM resveratrol for 3 days or with 10 μM resveratrol for 7 and 9 days exhibited statistically lower intracellular concentrations of IL1β compared with the control group (Figures 5B and C).

### Effect of resveratrol on the STAT3 signaling pathway in GMECs

**Figure 6:**
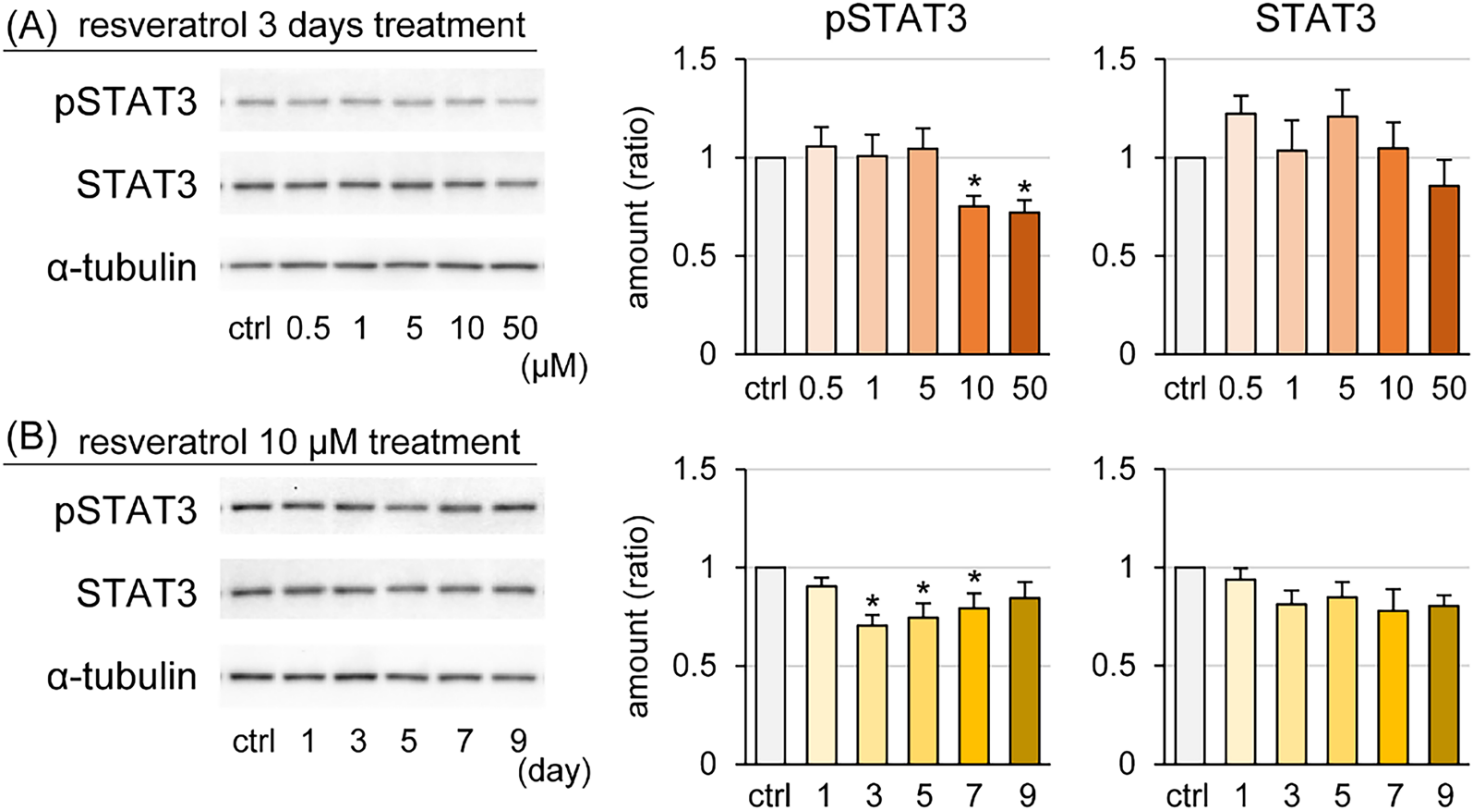
**Effect of resveratrol on STAT3 signaling in goat mammary epithelial cells (GMECs).** Western blot analysis of phosphorylated-STAT3 (pSTAT3) and STAT3 in GMECs treated with resveratrol at various concentrations for 3 days (**A**) or treated with 10 μM resveratrol each day (**B**). The graphs show the results of densitometry analysis; α-tubulin was the internal control. Control GMECs were treated with 0.1% dimethyl sulfoxide. The data are presented as the mean ± SEM (*n* = 8). Asterisks show significant differences (*p* < 0.05 versus control).

The influence of resveratrol on the STAT3 signaling pathway was examined by Western blot analysis (Figure 6). GEMCs treated with resveratrol at 10 or 50 μM for 3 days significantly decreased the amount of pSTAT3 to less than 75% of that in the control group (Figure 6A). Moreover, GEMCs treated with 10 μM resveratrol for 3, 5, and 7 days also significantly decreased pSTAT3 levels compared with the control group (Figure 6B). In contrast, there was no statistical effect on the amount of STAT3 protein.

## Discussion

Claudin-3 is responsible for less-permeable TJs in MECs during lactation [4]. Resveratrol increased the total amount of claudin-3 and the ratio of Triton X-insoluble/soluble claudin-3 in cultured GMECs. Claudins in the Triton X-100-insoluble fractions originate from the TJ regions [53]. In addition, resveratrol enhanced the transepithelial resistance of the MEC layer. Therefore, these results indicate that resveratrol strengthens the TJ barrier function with the upregulation of claudin-3. Moreover, topical application of resveratrol to lactating goat udders decreased the SCC, Na^+^, albumin, and IgG in milk, which are indicators of the blood-milk barrier [54]. IgG primarily leaks from the blood into milk through paracellular pathways [19]. Resveratrol treatment also decreased the concentration of cathelicidin-2 in milk, which is produced by leukocytes migrating into the mammary alveoli [24, 52]. These findings suggest that resveratrol treatment strengthens the blood-milk barrier resulting in decreased SCC, Na^+^, albumin, IgG, and cathelicidin-2 in milk.

Antimicrobial compounds in milk are important for host defense against pathogens that invade mammary glands [5]. Resveratrol promoted the production of β-defensin-1, S100A7, and lactoferrin in cultured GMECs and enhanced the secretion of β-defensin-1 and S100A7 into the medium. In addition, topical application also significantly increased β-defensin-1 and S100A7 levels in milk. The main mastitis induced pathogens of cows and goats are *E. coli*, *Staphylococcus aureus*, Coagulase-negative *Staphylococci*, *Streptococci*, and *Klebsiella pneumoniae* [55–57]. S100A7 exhibits antimicrobial activity against *E. coli* [17] and β-defensin-1 exerts antibacterial activity against a broad spectrum of microorganisms [14]. Thus, it is expected that an increase in antimicrobial compounds in milk represses the growth or viability of mastitis-causing pathogens. In addition, some mastitis-causing pathogens invade bovine MECs [58]. An increase in the intracellular concentration of antimicrobial compounds may provide an effective defense against pathogens. Taken together, we demonstrate that the topical application of resveratrol to udders enhances the production of antimicrobial compounds.

Resveratrol decreased the amount of phosphorylated STAT3 in GMECs. During inflammation or after weaning, the STAT3 pathway is activated in MECs, resulting in the induction of cytokine production, disruption of the TJ barrier, and decreased milk production [50, 59, 60]. Resveratrol also decreased IL1β levels in cultured GMECs and milk, which in turn, decreases the production of milk components [61] and disrupts the TJ barrier in MECs [62]. In addition, treatment using resveratrol temporarily decreased IL8 levels in milk. IL8 is a chemotactic factor of neutrophils [63], which may be involved in a decrease in SCC by resveratrol. Therefore, resveratrol may indirectly regulate TJ barrier function and antimicrobial compound production through STAT3 signaling and cytokine production.

Resveratrol protects bovine MECs from oxidative damage induced by H_2_O_2_ by regulating antioxidant activity [28]. In MECs, high milk production generates reactive oxygen species, which causes oxidative stress [64]. The accumulation of oxidative stress also induces cytokine production, TJ barrier weakness, and decreased milk production [65–67]. Resveratrol may regulate the induction of endogenous oxidative stress. Further studies are needed to determine the relationship between oxidative stress induced by milk production and antioxidant activity regulated by resveratrol in lactating MECs.

In conclusion, we determined the effect of resveratrol on TJ barrier and antimicrobial compound production in GMECs. Resveratrol promoted the secretion of β-defensin-1 and S100A7 and enhanced TJ barrier function with an increase in claudin-3 levels. In addition, resveratrol repressed cytokine production and STAT3 signaling in GMECs. Furthermore, topical application of resveratrol to goat udders produced the same effects as in vitro experiments. Thus, our findings suggest that resveratrol application may be effective at preventing mastitis by enhancing TJ barrier function and antimicrobial compound production in mammary glands. These findings may contribute to the effective use of by-products or food loss and waste derived from wine production. Moreover, the oral administration of polyphenols results in reduced biological activity through conjugation and metabolic conversion, although several polyphenols can regulate certain processes in cultured MECs [46, 68, 69]. The results of this study may provide insight into new uses of polyphenols in mammary glands and other tissues. This study did have some limitations, such as the small sample size and some individual differences in the effects; therefore, further studies should use a greater number of animals, including dairy cows.

### Supplementary Information

**Additional file 1. Effect of a topical application of 0.2% dimethyl sulfoxide to udders on milk yield and somatic cell count (SCC), blood-derived components, antimicrobial compounds, and cytokine levels in milk.** The udders of lactating goats were topically treated with 1 mL of 70% ethanol containing 0.2% dimethyl sulfoxide for 7 days. The graphs show changes in milk yield, SCC, Na^+^, albumin, IgG, β-defensin-1, S100A7, lactoferrin, cathelicidin-2, S100A8, IgA, IL1β, TNFα, and IL8 levels. The relative values compared with the average of pre-treated (days −1 and 0) controls. The data are presented as the mean ± SEM (*n* = 9).

## Abbreviations

DMEM/F12: Dulbecco Modified Eagle Medium/Ham’s F-12
DMSO: dimethyl sulfoxide
ELISA: enzyme-linked immunosorbent assay
GMEC: goat mammary epithelial cell
HBSS: Hanks Balanced Salt Solution
HRP: horseradish peroxidase
IL: interleukin
MEC: mammary epithelial cell
PCR: polymerase chain reaction
SCC: somatic cell count
TJ: tight junction
TNF: tumor necrosis factor

## Declarations

### Data Availability

The original contributions presented in the study are included in the article/supplementary material, further inquiries can be directed to the corresponding author.
