# Supplementary material for: Effects of topical application of resveratrol on tight junction barrier and antimicrobial compound production in lactating goat mammary glands
Source: Vet Res. 2024 Feb 16;55:20. doi: 10.1186/s13567-024-01276-z (PMC10870570; doi:10.1186/s13567-024-01276-z)
Supplement: Supplementary file 1 [file 13567_2024_1276_MOESM1_ESM.docx]

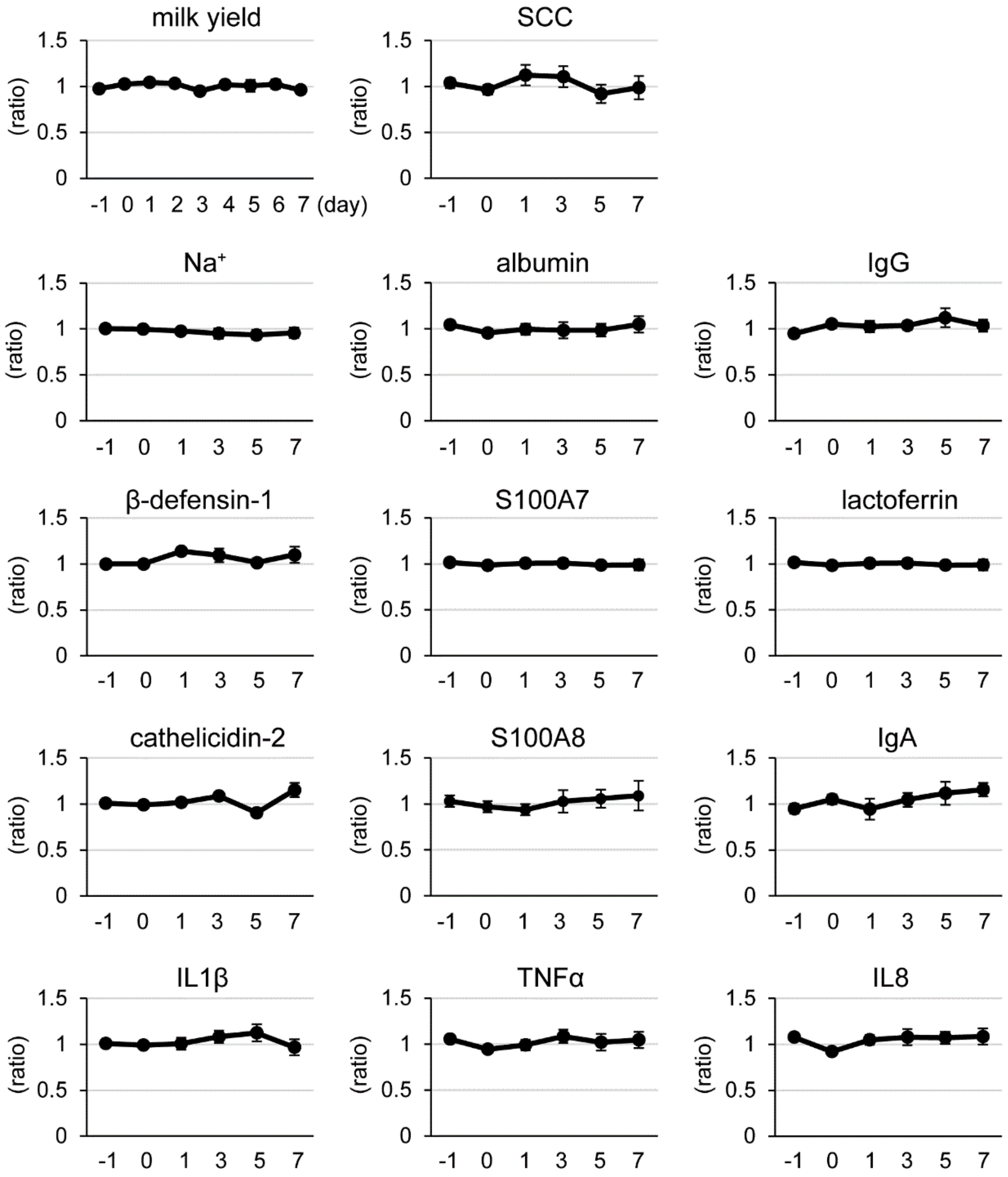
milk yield
SCC
Na⁺
albumin
IgG
β-defensin-1
S100A7
lactoferrin
cathelicidin-2
S100A8
IgA
IL1β
TNFα
IL8
(ratio)
1.5
1
0.5
0
-1 0 1 2 3 4 5 6 7 (day)
-1 0 1 3 5 7
